# A Subtle Adversary: Mycobacterium avium-intracellulare Infection of the Surgical Site Years After Bilateral Lung Transplantation

**DOI:** 10.7759/cureus.72760

**Published:** 2024-10-31

**Authors:** Andrew J Gorton, Suresh Keshavamurthy, Michael I Anstead

**Affiliations:** 1 Department of Surgery, Division of Cardiothoracic Surgery, University of Kentucky, Lexington, USA; 2 Department of Surgery, Division of Cardiothoracic Surgery, University of Texas Southwestern Medical Center, Dallas, USA; 3 Department of Medicine, Division of Pulmonology, University of Kentucky, Lexington, USA

**Keywords:** cutaneous infection, immunosuppression, lung transplant, medical therapy, mycobacterium avium

## Abstract

*Mycobacterium avium-intracellulare* complex is a nontuberculous *Mycobacterium* that has been associated with a wide range of infections, including the skin and soft tissues, central nervous system, and pulmonary system. We discuss the case of a 66-year-old male with a history of coal worker’s pneumoconiosis who had undergone a bilateral lung transplant eight years prior and presented with drainage from his right chest incision site. He underwent operative exploration and drainage of the wound. Cultures returned with *M. avium-intracellulare* complex. He was initially treated with rifabutin, ethambutol, and azithromycin for a planned 12-month course. Ethambutol was subsequently discontinued due to a suspected drug fever. At this time, he is recovering appropriately and doing well on his antibiotic course. While still a rare cause of infection within the *Mycobacterium* genus, it has been increasingly recognized. Recognition is important due to the antimicrobial drug-resistant characteristics of nontuberculous​​​​​​​ *Mycobacterium*. They have also been found to be resistant to disinfectants, making them an important source of postsurgical infection. Skin and soft tissue infections are commonly associated with direct contact with contaminated material and an open wound or secondary to disseminated disease. Susceptibility testing is a necessity to determine the appropriate treatment. Clarithromycin, amikacin, and cefoxitin have shown the best activity in in vitro studies. Prevention of contamination via control of the water supply and medical devices is an important measure. This case demonstrates that nontuberculous​​​​​​​ *Mycobacterium* infections may present in at-risk patients remotely following surgery and require a high level of clinical suspicion for diagnosis and treatment.

## Introduction

*Mycobacterium avium-intracellulare* complex (MAC) is a nontuberculous *Mycobacterium* (NTM) that has been associated with a wide range of infections, including the skin and soft tissues, central nervous system, and pulmonary system [1]. We discuss the case of a 66-year-old male with a history of coal worker’s pneumoconiosis who had undergone a bilateral lung transplant eight years prior and presented with drainage from his left chest incision site. He underwent operative exploration and drainage of the wound. Cultures returned with *M. avium-intracellulare*. He was initially treated with rifabutin, ethambutol, and azithromycin for a planned 12-month course. Ethambutol was subsequently discontinued due to a suspected drug fever. At this time, he is recovering appropriately and doing well on his antibiotic course. While still a rare cause of infection within the *Mycobacterium* genus, it has been increasingly recognized [2]. Recognition is important due to the antimicrobial drug-resistant characteristics of NTM. They have been found to be resistant to disinfectants, making them an important source of postsurgical infections. Skin and soft tissue infections are commonly associated with direct contact with contaminated material and an open wound or secondary to disseminated disease. Importantly, NTM is resistant to standard antituberculous agents and many other antimicrobials. Susceptibility testing is a necessity to determine the appropriate treatment. Clarithromycin, amikacin, and cefoxitin have shown the best activity in in vitro studies. Prevention of contamination via control of the water supply and medical devices is an important measure.

## Case presentation

**Figure 1:**
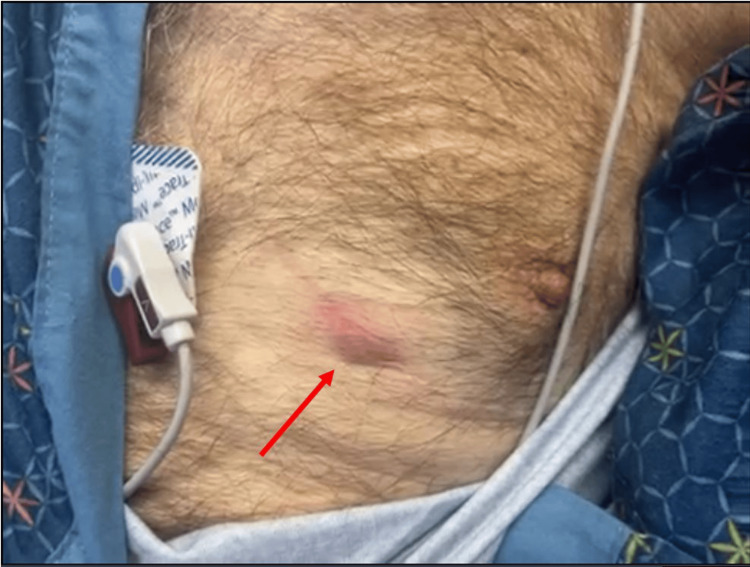
The preoperative wound on the left chest at the site of the previous clamshell sternotomy incision (marked with a red arrow).

**Figure 2:**
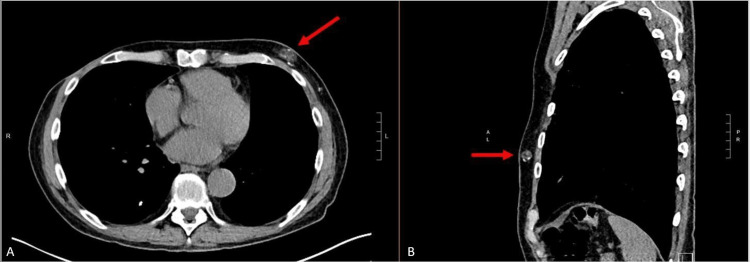
Preoperative computed tomography scan of the chest without contrast showing nodularity of the left chest wall (marked with red arrows). Left (A) shows the axial view. Right (B) shows the sagittal view.

**Figure 3:**
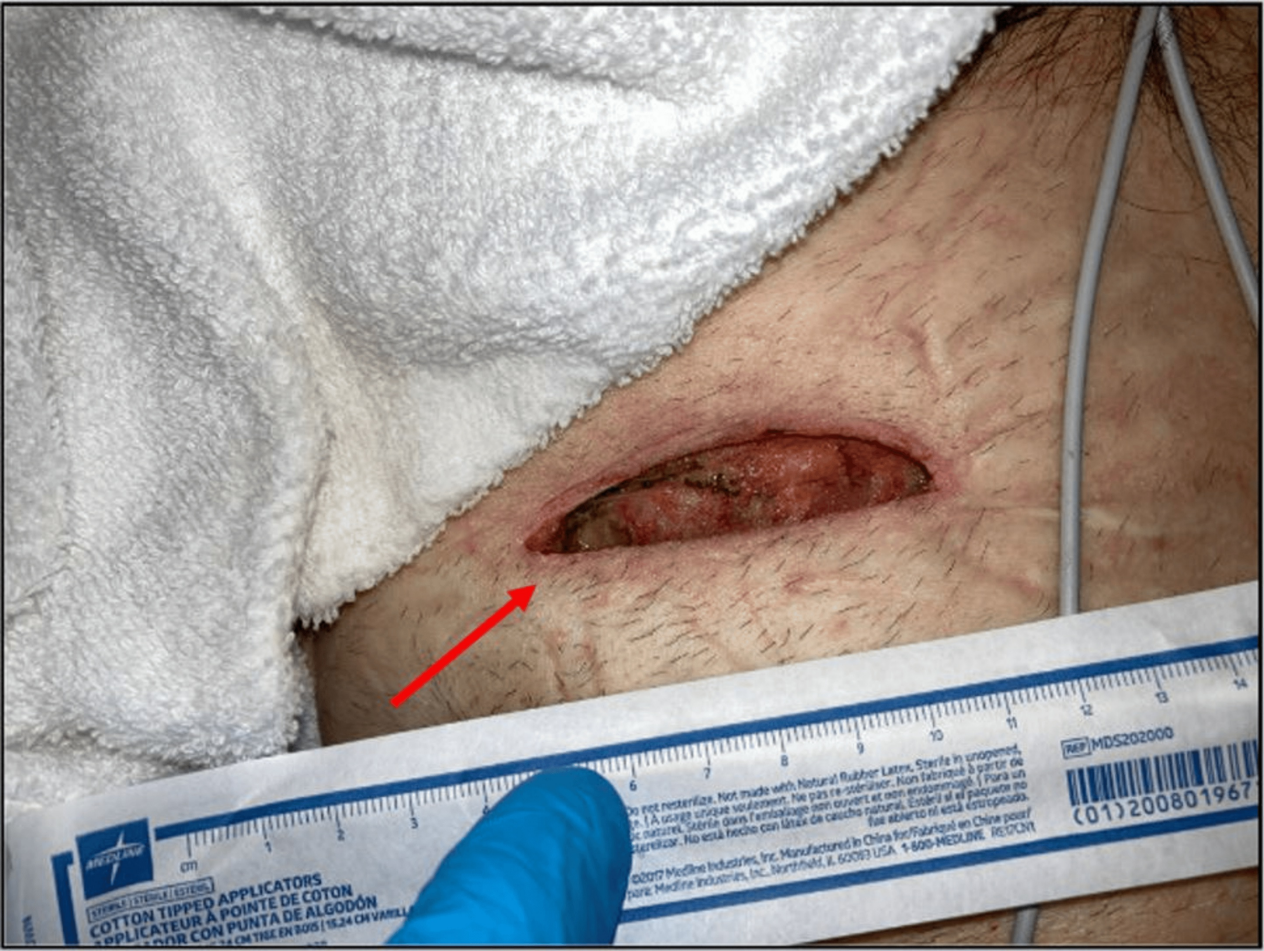
Left chest wound following debridement (marked with a red arrow).

We present the case of a 66-year-old male with a history of coal worker’s pneumoconiosis who had undergone bilateral lung transplantation eight years prior and presented with drainage from the site of his left chest incision (Figure 1). He was otherwise in normal health and denied any recent trauma or illness. A computed tomography scan of the chest did not identify any discrete inflammation or fluid collection (Figure 2). Still, the decision was made to pursue operative exploration. He underwent exploration of the draining sinus, excisional debridement, removal of sternal wires, and wound irrigation. This revealed a large well-encapsulated “yellow-curd” collection. The wound was irrigated with hydrogen peroxide and antibiotic saline (gentamicin and amphotericin). The final wound dimensions were 2.5 x 10 cm superficially, 1.5 cm deep, and 5 x 16 cm at the wound base. Local wound care was continued with every other day wound vac changes (Figure 3).

Collected cultures were positive for acid-fast bacilli (AFB) stain and revealed MAC. It is our institutional practice to send all intraoperative specimens for broad culture evaluation to include aerobic, anaerobic, and fungal organisms. At this time, he was started on rifabutin (150 mg daily), ethambutol (1200 mg Monday-Wednesday-Friday), and azithromycin (250 mg daily) for a planned 12-month course. Intraoperative cultures were also positive for *Propionibacterium* and *Corynebacterium* for which he received an eight-week course of oral penicillin V potassium. At this admission, his pulmonary graft function was stable on the basis of pulmonary function testing with a forced expiratory volume in one second (FEV1) of 1.77 liters (52% predicted). He remained on standard immunosuppression with mycophenolate mofetil, tacrolimus, and prednisone. Ongoing antimicrobial prophylaxis was continued with sulfamethoxazole-trimethoprim and valganciclovir.

His initial postoperative course was uncomplicated, and he was discharged home shortly after the debridement. He was readmitted approximately one month later with a high-grade fever. Re-evaluation did not identify any new source of infection and it was determined that he was experiencing medication-induced hyperthermia related to ethambutol administration. He was continued on azithromycin and rifabutin. As of his one-year follow-up, his wound has healed and he has no further evidence of MAC infection. Bronchoscopic evaluation at that time revealed normal surgical anastomoses and graft tracheobronchial tree to the subsegmental level. Spirometry showed relatively unchanged graft function with an FEV1 of 1.48 liters (45% predicted). He remains on a standardized post-transplant surveillance with scheduled tissue sampling and bronchoalveolar lavage.

## Discussion

MAC is a slow-growing NTM with many species, including *M. avium*, *M. intracellulare*, *M. chimaera*, and many more. Localized extrapulmonary MAC infection of the skin and soft tissues is a rare entity without well-established treatment guidelines. The American Thoracic Society’s published guidelines acknowledge that beyond surgical excision or debridement, there is little data to drive therapeutic choices [3]. Standard therapy tends to consist of six to 12 months of a three-drug antimicrobial regimen with azithromycin or clarithromycin, rifampin or rifabutin, and ethambutol [4]. The most common modes of acquiring cutaneous MAC infections are trauma, cosmetic procedures, and postsurgical infections [1]. They present with a variety of lesions, including papular, verrucous ulcers, inflammatory pseudotumors, draining sinuses, and cold abscesses [2].

MAC has varying ubiquitousness within NTM infections based on geographic region with the highest prevalence being found in North America and Europe where MAC represents around 50% of NTM infections [5]. Diagnosis may be complicated by the clinical similarity to *M. tuberculosis* and other NTMs. One of the primary methods for diagnosis is AFB staining on smear microscopy. Unfortunately, this testing is of low sensitivity and does not characterize the *Mycobacterium*. Molecular testing is often necessary for *Mycobacterium* speciation determinations. The expedient diagnosis we obtained in our patient is not often seen. Another diagnostic challenge is that many patients are colonized with *Mycobacterium* that are not contributing to active illness. Guidelines related to pulmonary infection are more well-defined than in patients with disseminated or extrapulmonary disease, but the antimicrobial regimen is similar.

NTM infections have long been described in immunocompromised patients, including those with cancer, transplant recipients, and human immunodeficiency virus (HIV). Around half of these NTM infections are pulmonary, one-quarter are skin and soft tissue infections, and one-quarter are disseminated diseases [6]. Within the lung transplant population, pulmonary NTM infections are of great concern, but relatively rare. In one series of lung transplant patients undergoing surveillance bronchoalveolar lavage (BAL), the rate of mycobacterial infection was 3.8% with the majority of cases of *Mycobacterium* in the native lung of single lung transplant recipients. In this series, the patients with pulmonary NTM were asymptomatic and treated appropriately [7]. Another reported series of lung transplant recipients found a 1.74% rate of *M. abscessus* infection based on respiratory cultures. Most cases were identified within the first year following transplant and underwent antimicrobial treatment. There was a high rate of treatment failure (44.4%) of treated patients defined by airway complications, dissemination of disease, and one death [8]. While genotypically distinct from MAC, this demonstrates the difficulty in the treatment of NTMs in lung transplant recipients. Another potential source for infection in heart and lung transplant recipients is the use of cardiopulmonary bypass (CPB) or extracorporeal membrane oxygenation (ECMO). Multiple reports have identified NTM infections within components of these circuits with water circuits in the heater-cooler units commonly identified as a source. While these are often fast-growing NTMs, *M. chimaera* of the MAC family was linked to endocarditis and bloodstream infections in six patients following cardiac surgery [9]. Focal pulmonary MAC has been treated surgically with lung resection. One such case report describes a delayed surgical site infection with MAC following lung resection that required further debridement and subsequent antimicrobial treatment [10].

Our case involves an immunosuppressed lung transplant patient well removed from his initial surgery. He was at high risk for NTM infections at baseline due to his immunosuppressive regimen. However, his operation was over eight years prior, and he had no recent trauma, illnesses, or history of known MAC colonization. He was treated with a combination of surgical debridement, local wound care, and multi-drug antimicrobial therapy with apparent resolution of his infection at one year. At this point, he only remained on his transplant prophylactic regimen. This case required a multidisciplinary approach between transplant surgery, pulmonology, and infectious disease teams among others. We feel this case is notable due to the timeline of infection of a surgical site well beyond the index operation without any evidence of prior colonization with the slow-growing MAC. The literature does not suggest any routine surveillance for NTMs in lung transplant recipients due to the rare nature of infections but does show that these infections can be serious when developed.

## Conclusions

MAC is an NTM that has been associated with a wide range of infections, including the skin and soft tissues, central nervous system, and pulmonary system. Lung transplant recipients and other immunocompromised patients are at higher risk of infection with MAC and other NTMs. Treatment is based on the disease process and specific NTM isolated but may include a combination of surgical intervention and medical therapy. The case presented shows that MAC surgical site infection may present many years following surgery. A multidisciplinary approach is key to timely diagnosis and treatment. The specific treatment regimen will need to be tailored to the patient and the species of NTM identified. In our case, following treatment of the acute infection, the patient returned to a standard post-transplant evaluation and prophylactic regimen.
